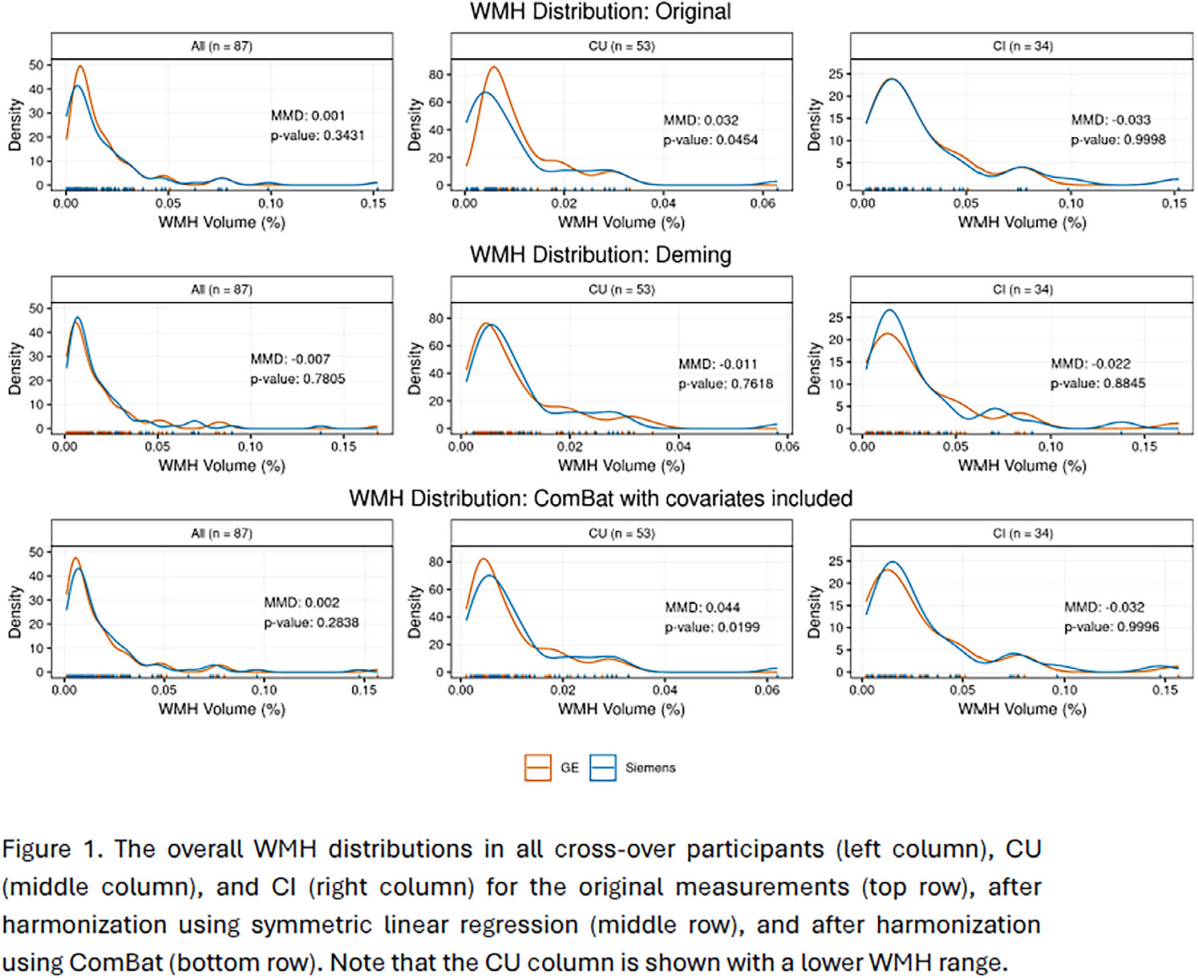# Harmonization Remains Unresolved: The Case of White Matter Hyperintensity Using ComBat And Deming Regression

**DOI:** 10.1002/alz70861_108630

**Published:** 2025-12-23

**Authors:** Calvin D. Reyes, Robel K Gebre, Jeffrey L. Gunter, Mingzhao Hu, Christopher G Schwarz, Nolan K. Meyer, Robert I. Reid, Kejal Kantarci, Clifford R. Jack, Prashanthi Vemuri

**Affiliations:** ^1^ Mayo Clinic, Rochester, MN USA; ^2^ Department of Radiology, Mayo Clinic, Rochester, MN USA; ^3^ Department of Quantitative Health Sciences, Mayo Clinic, Rochester, MN USA

## Abstract

**Background:**

Multi‐site aging and dementia studies are challenged by data heterogeneity caused by scanner (manufacturer and protocol) related variability. Quantitative analysis of white matter hyperintensity (WMH) from pooled samples, a traditional marker of cerebrovascular disease, is sensitive to machine variability. Our goal was to compare Deming (symmetric) linear regression and ComBat, a common method used for harmonizing neuroimaging measurements, for harmonizing WMH measurements on participants scanned on two different scanners (GE and Siemens) and protocols (2D FLAIR on GE and 3D FLAIR on Siemens).

**Methods:**

Cognitively unimpaired (CU) (*n* =53, age (mean (SD))=67 (15) years) and cognitively impaired (CI; MCI/dementia) (*n* =34, age (mean (SD))=82 (9) years) participants from the Mayo Clinic Study of Aging and Mayo ADRC were scanned on GE and Siemens scanners within a week of each other (typically done on the same day). WMH as a percentage of total intracranial volume was measured using multispectral segmentation of FLAIR and T1‐weighted images (Gunter et. al. AAIC 2023). We used Deming regression as it includes errors in both input dimensions. ComBat was applied using age, sex, and diagnosis as covariates, with scanner vendor and protocol as the batch variable. We assessed the inter‐scanner similarity using Maximum Mean Discrepancy (MMD) analysis, where a value of zero indicates identical distributions, *p* >0.05 indicates no significant difference under H_0_. (Gretton et al., JMLR 2012).

**Results:**

Our results showed that the distribution of WMH measurements, which were different in the CU group, were more similar after Deming than ComBat harmonization (Figure 1). This was confirmed by the lower MMD in the CU group from Deming regression (‐0.01 (*p* =0.76) from 0.03 (*p* =0.05)) compared to ComBat (0.04 (*p* =0.02))(Figure 1). However, ComBat and Deming were unable to create distributions as compatible as original measurements for the CI group.

**Conclusion:**

Using a unique dataset of the same participants scanned close in time on different scanner and protocols, we found that (1) MMD measurements were useful to test the need/improvement due to harmonization; (2) ComBat was not substantially better at creating compatible distributions between scanner vendors than Deming regression, and both were sometimes inadequate. Future work will require alternative models to linear scale and shift transformations.